# Mesenchymal Stem Cell Therapy Facilitates Donor Lung Preservation by Reducing Oxidative Damage during Ischemia

**DOI:** 10.1155/2019/8089215

**Published:** 2019-08-05

**Authors:** Natalia Pacienza, Diego Santa-Cruz, Ricardo Malvicini, Oscar Robledo, Gastón Lemus-Larralde, Alejandro Bertolotti, Martín Marcos, Gustavo Yannarelli

**Affiliations:** ^1^Laboratorio de Regulación Génica y Células Madre, Instituto de Medicina Traslacional, Trasplante y Bioingeniería (IMeTTyB), Universidad Favaloro-CONICET, Solís 453, CABA (1078), Buenos Aires, Argentina; ^2^Departamento de Cirugía, Facultad de Ciencias Veterinarias, Universidad Nacional de La Plata, Calle 60 y 118, La Plata (1900), Buenos Aires, Argentina; ^3^Departamento de Cirugía Cardiovascular y Torácica, Hospital Universitario Fundación Favaloro, Av. Belgrano 1746, CABA (1039), Buenos Aires, Argentina

## Abstract

Lung transplantation is a lifesaving therapy for people living with severe, life-threatening lung disease. The high mortality rate among patients awaiting transplantation is mainly due to the low percentage of lungs that are deemed acceptable for implantation. Thus, the current shortage of lung donors may be significantly reduced by implementing different therapeutic strategies which facilitate both organ preservation and recovery. Here, we studied whether the anti-inflammatory effect of human umbilical cord-derived mesenchymal stem cells (HUCPVCs) increases lung availability by improving organ preservation. We developed a lung preservation rat model that mimics the different stages by which donor organs must undergo before implantation. The therapeutic schema was as follows: cardiac arrest, warm ischemia (2 h at room temperature), cold ischemia (1.5 h at 4°C, with Perfadex), and normothermic lung perfusion with ventilation (Steen solution, 1 h). After 1 h of warm ischemia, HUCPVCs (1 × 10^6^ cells) or vehicle was infused via the pulmonary artery. Physiologic data (pressure-volume curves) were acquired right after the cardiac arrest and at the end of the perfusion. Interestingly, although lung edema did not change among groups, lung compliance dropped to 34% in the HUCPVC-treated group, while the vehicle group showed a stronger reduction (69%, *p* < 0.0001). Histologic assessment demonstrated less overall inflammation in the HUCPVC-treated lungs. In addition, MPO activity, a neutrophil marker, was reduced by 41% compared with vehicle (*p* < 0.01). MSC therapy significantly decreased tissue oxidative damage by controlling reactive oxygen species production. Accordingly, catalase and superoxide dismutase enzyme activities remained at baseline levels. In conclusion, these results demonstrate that the anti-inflammatory effect of MSCs protects donor lungs against ischemic injury and postulates MSC therapy as a novel tool for organ preservation.

## 1. Introduction

Lung transplantation has become the standard treatment for end-stage respiratory diseases. Although there is an increase in the number of annual lung transplants, the mortality on current waiting lists can be as high as 30% due to the shortage of organs available for transplantation [1, 2]. Several factors are responsible for this deficit. First, there is a low number of multiorgan brain death donors available. Second, only about 15% of lungs from these donors are suitable for transplantation as most of them are discarded due to injuries caused by brain death and/or poor donor management [3]. Third, transplant programs are usually very conservative in the selection of donor lungs to avoid severe complications associated with primary graft dysfunction (PGD). PGD is the end result of a series of injuries occurring in the donor organ from the time of death to reperfusion in the recipient [4]. Hence, the incidence of PGD is directly related to processes of sterile inflammation that occur in the lung transplantation setting, such as ischemia, ischemia-reperfusion injury (IRI), and mechanical ventilator-induced injury [5]. All these processes cause an increase in the production of reactive oxygen species (ROS) and the consequent cellular damage. Thus, oxidative stress plays a key role in the development of donor lung injury, which is mainly characterized by edema, ineffective gas exchange, increased levels of proinflammatory cytokines, and pulmonary infiltrates [6, 7]. In fact, early neutrophil extravasation into the alveolar space and the formation of neutrophil extracellular traps produce lung damage in both IRI and PGD [8]. Recent data suggest that neutrophil recruitment is mediated by ROS-activated alveolar macrophages and monocytes [9]. In addition, ROS can activate antigen-presenting cells that trigger the adaptive immune response leading to organ rejection [10]. Thus, the strong association between PGD and lung allograft rejection may be linked by ROS and alveolar macrophages [11].

In order to solve the existing organ shortage, it is imperative not only to increase the organ procurement, but also to ensure the good quality of the retrieved lungs by adopting new therapeutic approaches which better preserve those organs. At present, several strategies are being used, including improvement in donor lung preservation, use of organs from donors with extended/marginal characteristics such as cardiac death donors (DCD), and normothermic ex vivo lung perfusion (EVLP) to assess and repair injured donor lungs [12]. Some of these strategies may also benefit from the use of gene and stem cell therapy, which have demonstrated therapeutic potential for solid organ graft optimization. In this context, human interleukin- (IL-) 10, a potent anti-inflammatory cytokine, has been shown to reduce IRI and improve graft function when administered through the transtracheal route prior to donor lung retrieval [13]. More recently, IL-10 gene therapy delivered in an EVLP system demonstrated improved lung function in a pig model of lung transplantation [14]. Similarly, mesenchymal stem cell (MSC) therapy has been shown to be safe and effective for the recovery of damaged lungs when administered during EVLP [15, 16] and also for the treatment of IRI in kidney, liver, heart, and lung transplantation [17–19]. Additionally, MSC therapy was able to reduce inflammatory responses in a mouse model of LPS-induced acute lung injury [20]. However, whether MSC therapy improves donor lung preservation has not been previously studied. As MSCs demonstrated immunomodulatory and anti-inflammatory properties, we hypothesized that the administration of MSCs during the warm ischemia period may better preserve the donor organ by preventing lung inflammation and limiting, in consequence, further injuries related to ischemia and reperfusion.

The umbilical cord has become an attractive and readily available source of MSCs [21]. Human umbilical cord perivascular cells (HUCPVCs) are isolated from neonatal tissue and present higher levels of stromal progenitors and proliferation capacity than bone marrow-derived MSCs [22]. Thus, HUCPVCs are being used as a rich source of MSCs for cell replacement therapy in preclinical and, more recently, clinical studies [21]. In fact, HUCPVCs demonstrated improved regenerative properties for acute myocardial infarction [23] and bone repair [24]. In this study, we investigated whether the local infusion of HUCPVCs during donor lung ablation increases lung availability by improving organ preservation. For this purpose, we developed a donor lung preservation rat model that mimics the different phases by which donor organs must undergo before implantation. We found that HUCPVCs protect donor lungs against ischemic injury by reducing oxidative damage and neutrophil extravasation into the lung tissue. More importantly, HUCPVC therapy significantly conserved lung function during ischemia and, thus, represents a novel tool for donor lung preservation.

## 2. Materials and Methods

### 2.1. HUCPVC Culture

HUCPVCs were gently provided at passage 2 by Dr. Mazzolini (Laboratory of Gene Therapy, IIMT, CONICET-Universidad Austral). HUCPVCs were cultured in DMEM low-glucose medium supplemented with 10% (v/v) of fetal bovine serum (FBS, Gibco), 100 U/ml penicillin, and 100 *μ*g/ml streptomycin (Gibco) at 37°C in a humidified incubator containing 5% CO_2_. The medium was replaced every 2–3 days, and HUCPVCs were subcultured at 70–90% confluence until passage 4-6 cells were obtained [23]. HUCPVCs were characterized according to the International Society for Cellular Therapy (ISCT) guidelines [25]. MSC surface-specific markers (CD14, CD34, CD44, CD73, CD90, and CD105 all from BD Biosciences) were determined by flow cytometry (FACSCalibur flow cytometer, Becton Dickinson), and data acquired were analyzed using FlowJo software (Tree Star).

### 2.2. Donor Lung Preservation Rat Model

All the procedures conformed to the US National Institute of Health's guidelines for the care and use of laboratory animals with approval from the Animal Care and Use Committee of the Favaloro University (CICUAL-UF). Twelve-week-old male Wistar rats (285 ± 27 g) were obtained from Facultad de Ciencias Veterinarias, Universidad Nacional de La Plata. Anesthesia was induced using an intraperitoneal injection of 60 mg/kg ketamine and 5 mg/kg xylazine. Heparin (1000 U/kg) was administered intrahepatically and allowed to circulate for 5 min. Animals were then euthanized with an intrahepatic injection of sodium thiopental solution (250 mg/kg). Following cardiac arrest (time = 0), an endotracheal tube was secured in the trachea and median sternotomy was performed. Lungs were mechanically ventilated (SomnoSuite, Kent Scientific) to acquire the basal pressure-volume curve. Lungs were not flushed to remove blood and were left untouched in the donor for 45 min. Then, the entire heart-lung block was excised, a cannula was placed in the pulmonary artery (PA), and the left atrium (LA) was opened (LA pressure = 0 cm H_2_O). At this point (1 h), lungs were suspended in a humid chamber at room temperature and randomly divided into two groups: vehicle control, receiving 1 ml of Krebs-Henseleit bicarbonate buffer containing 4% bovine serum albumin, or HUCPVCs, receiving cell therapy (1 × 10^6^ cells in 1 ml Krebs-Henseleit solution) administered by using a 1 ml syringe via the PA. After the treatment, lungs were kept at room temperature for another hour. In total, lungs underwent 2 h of warm ischemia without mechanical ventilation. Lungs were then infused via the PA (PA pressure < 15 cm H_2_O) with 10 ml of cold preservation solution (Perfadex) and kept at 4°C for 90 min. After the cold ischemia period, ungs were rewarmed to room temperature and perfused through the PA with Steen solution alone for 1 h (PA pressure < 15 cm H_2_O, perfusion rate = 2 ml/min). The temperature of the perfusate was gradually increased from room temperature to 37°C for approximately over 30 min. The perfusion was continued at 37°C until reaching 60 min. During EVLP, lungs were mechanically ventilated starting with a short alveolar recruitment strategy which included a gradual increase in both the positive end-expiratory pressure (PEEP) up to 8 cm H_2_O and the respiratory rate from 20 to 60 breaths per min. Ventilation was continued using a peak airway pressure (PAWP) of 15 cm H_2_O, a PEEP of 2 cm H_2_O, and a respiratory rate of 60 breaths per min. Lung functional data (pressure-volume curve) were acquired at the end of the perfusion (60 min time point). A graphical guide of the surgical procedure is available as Supplementary Material (available here).

### 2.3. Pressure-Volume Curves and Lung Compliance Determination

Measurements were performed in the isolated lungs by using a small animal mechanical ventilator (SomnoSuite, Kent Scientific) (Figure S1). The pressure-volume (P-V) curves were obtained using the regular pressure ramp approach up to a maximal tidal volume of 10 ml/kg to avoid lung overdistension. Briefly, lungs were inflated to a given pressure (PAWP of 4, 8, 12, and 16 mm H_2_O were applied), and the tidal volume reached at each pressure step was measured. This method is based on the single-compartment linear model, in which the P-V relationship corresponds to a straight line and a single value describes the compliance. The tidal volume in ml/kg was plotted against the pressure difference between the PAWP and PEEP. Lung compliance was calculated from the slope of the linear regression model [26].

### 2.4. CFSE Cell Staining and Flow Cytometry

HUCPVCs were harvested, resuspended in PBS at 2 × 10^6^ cells/ml, and incubated with 2.5 *μ*M carboxyfluorescein diacetate succinimidyl ester (CFSE, eBioscience) for 5 min. Cells were then washed and resuspended in culture medium for 5 min at 37°C to stabilize the CFSE staining. After a final wash step, cells were plated at a density of 4,000 cells/cm^2^ and cultured for 48 h. CFSE staining was confirmed by using fluorescence microscopy and flow cytometry. To determine the presence of donor cells in the lungs, CFSE-labeled HUCPVCs were infused through the pulmonary artery as described above (see Section 2.2). At the end of the experiment, samples were obtained from different sections of the left lung (upper, middle, and lower sections). Lung samples were then minced with scalpels, incubated in Hank's balanced salt solution containing 1 mg/ml collagenase type 2 (Worthington Biochemical Corp.) for 30 min, and passed through 40 *μ*m nylon mesh to obtain a single-cell suspension. Data from three independent experiments were acquired in a FACSCalibur flow cytometer (Becton Dickinson) and analyzed using FlowJo software (Tree Star).

### 2.5. Histological Examination and Immunostaining

For histopathological examination, lung biopsies were taken at the end of the perfusion. Lung tissue was fixed in 10% neutral-buffered formaldehyde for 48 h, embedded in paraffin, and sectioned. Sections (5 *μ*m thick) were stained with hematoxylin-eosin and examined at a ×200 magnification. To determine the extent of lung injury, we considered (i) alveolar septal thickening, (ii) cellular infiltration, and (iii) atelectasis/collapse. Scoring was done by a pathologist who was blinded to the experimental groups. An injury score of 0–3 (0 = absent/none, 1 = mild, 2 = moderate, and 3 = severe) was assigned to each variable and used to calculate a total score for lung injury. A total score of 0 indicated normal histopathology and a total of 9 points indicated maximal damage. Immunohistochemical staining of paraffin-embedded lung sections was performed using an automated Ventana BenchMark GX instrument (Roche Diagnostics) according to the manufacturer's instructions. The presence of neutrophils was detected by using an anti-myeloperoxidase rabbit polyclonal antibody (Roche Diagnostics). To determine the presence of human donor cells in the lungs, we employed an anti-human vimentin (V9) mouse monoclonal antibody (Roche Diagnostics). Primary antibodies were detected by using the iVIEW DAB detection kit (Roche Diagnostics). Images were acquired using a Zeiss Axiophot microscope.

### 2.6. Quantification of Lung Wet/Dry Weight Ratio

Lung samples were immediately weighed and then placed in a desiccating oven at 55°C for 72 h; at that point, dry weight was measured. The ratio of wet/dry weight was used to quantify lung water content.

### 2.7. Myeloperoxidase Activity

MPO activity, an index of polymorphonuclear leukocyte accumulation, was determined as previously described [27]. Lung tissue samples (100-200 mg) were homogenized in 1 ml of a solution containing 0.5% hexa-decyl-trimethyl-ammonium bromide (HTAB) dissolved in 50 mM potassium phosphate buffer (pH 6). The suspension was sonicated and centrifuged for 30 min at 20,000g at 4°C. The supernatant was assayed for MPO activity. An aliquot of the supernatant (10 *μ*l) was combined with 190 *μ*l of 50 mM phosphate buffer (pH 6.0), containing 0.167 mg/ml o-dianisidine and 0.0005% H_2_O_2_. Changes in absorbance at 460 nm were measured spectrophotometrically. MPO activity is expressed as *μ*mol of degraded peroxide per minute per mg of protein at 25°C.

### 2.8. Protein Extracts

Tissue samples (100-200 mg) were suspended in 1 ml 50 mM potassium phosphate buffer (pH 7.4), containing 0.1% Triton X-100 and homogenized. Homogenates were sonicated for 20 s in an ice bath. Suspensions were centrifuged at 14,000g for 15 min, and supernatants were harvested. Protein concentrations were determined using the Bradford micromethod assay (Bio-Rad).

### 2.9. Protein Carbonyl Content

Protein carbonyl content was quantified using the 2,4-dinitrophenylhydrazine (DNPH) alkaline method [28]. Briefly, 80 *μ*l of DNPH (10 mM in 0.5 M H_3_PO_4_) was added to 80 *μ*l of protein solution, and the mixture was incubated for 10 min at room temperature. Forty *μ*l of NaOH (6 M) was then added. After 10 min of incubation at room temperature, absorbance was read at 450 nm against a blank in which the protein solution was substituted by an equal volume of buffer solution.

### 2.10. Antioxidant Enzyme Activities

Activities of the antioxidant enzymes superoxide dismutase (SOD) and catalase (CAT) were determined spectrophotometrically in tissue homogenates. SOD assays were performed using the riboflavin/nitroblue tetrazolium assay in a microtiter plate format [29]. Twenty *μ*l of lysate or SOD standard (0.5-500 U/ml) was measured in a final reaction mixture of 300 *μ*l potassium phosphate buffer (50 mM, pH 7.8) containing EDTA (0.1 mM), riboflavin (1.3 *μ*M), L-methionine (10 mM), p-nitro-blue tetrazolium (NBT,57 *μ*M), and Triton X-100 (0.025%). Absorbance was read at 560 nm both immediately and after 10 min incubation under a homogenous light field at 25°C. One unit of SOD activity was defined as the amount of enzyme able to inhibit the reduction of NBT by 50%. CAT activity was determined by measuring the decrease in light absorption at 240 nm in a reaction medium containing 50 mM potassium phosphate buffer (pH 7.2) and 2 mM H_2_O_2_ [30].

### 2.11. Statistics

Continuous variables were expressed as mean ± standard deviation (SD). One-way analysis of variance (ANOVA) with the post hoc Tukey's test was used for intergroup comparisons. Individual changes in lung compliance between baseline and perfusion were analyzed using paired *t* test. A value of *p* < 0.05 was considered statistically significant.

## 3. Results

### 3.1. Cell Therapy with HUCPVCs to Improve Donor Lung Preservation

**Figure 1:**
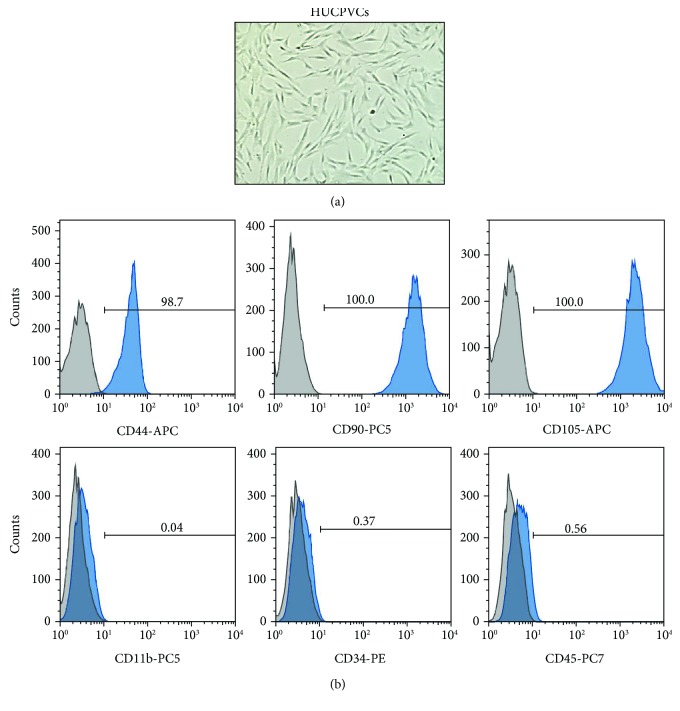
Characterization of human umbilical cord perivascular cells (HUCPVCs). (a) Representative phase micrograph image of HUCPVCs in culture. HUCPVCs display a fibroblastic morphology (×100 original magnification). (b) Surface marker expression levels in HUCPVCs analyzed by flow cytometry. HUCPVCs highly express the stromal determinants CD44, CD90, and CD105 and are negative for CD11b, CD34, and CD45 markers. Solid blue histograms represent cells stained with fluorescent antibodies, and isotype-matched controls are overlaid in gray.

**Figure 2:**
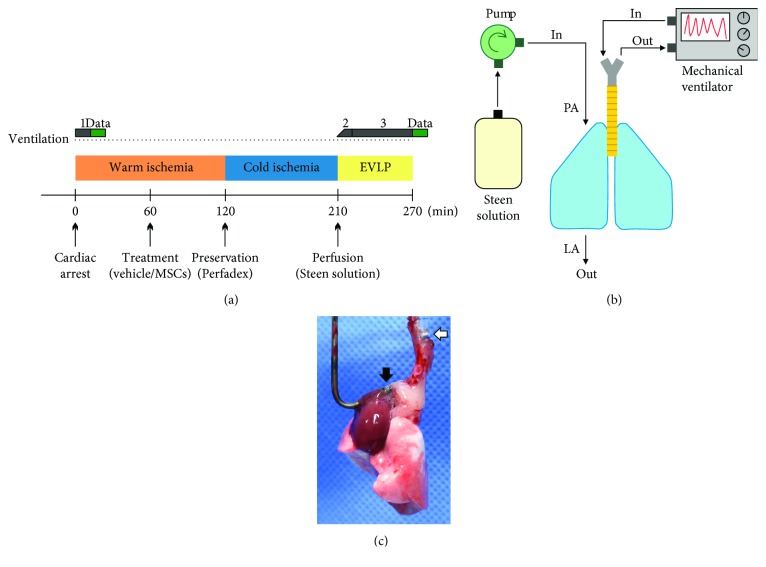
Donor lung preservation rat model. (a) Experimental study design. The procedure included cardiac arrest, warm ischemia, cold ischemia, and normothermic ex vivo lung perfusion using Steen solution with mechanical ventilation. Ablation of the lung was performed after 1 h of warm ischemia, and cell therapy was administered via the pulmonary artery. The lung was mechanically ventilated during perfusion starting with a short alveolar recruitment strategy (#2). Lung functional data were acquired right after the cardiac arrest (baseline, #1 green bar) and at the end of the perfusion (endpoint, #3 green bar). For further details, please refer to Materials and Methods (Section 2.2). (b) Schematic diagram of the normothermic ex vivo lung perfusion step. After the cold ischemia period, the lungs were rewarmed and perfused through the pulmonary artery (PA) with Steen solution for 1 h using an open perfusion circuit in which the left atrium (LA) was opened. During perfusion, the lungs were mechanically ventilated. (c) Representative image of a rat lung during perfusion. Please note the position of the PA cannula (black arrow) for the perfusion and the tracheotube (white arrow) for the mechanical ventilation.

**Figure 3:**
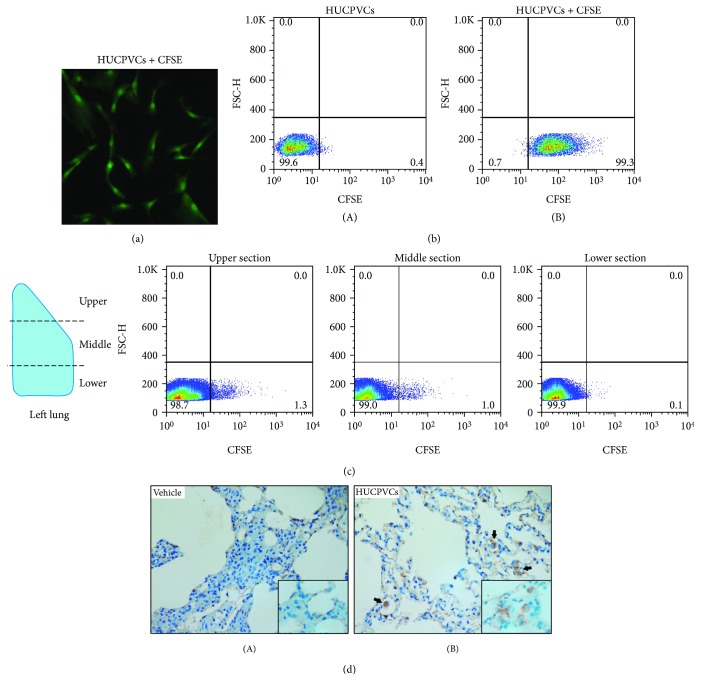
Retention of HUCPVCs in the lungs. (a) Representative image of CFSE-labeled HUCPVCs using fluorescence microscopy (×100 original magnification). (b) Flow cytometry analysis of CFSE-labeled HUCPVCs. (A) Dot plot for the unstained control. (B) Dot plot for CFSE-labeled HUCPVCs showing that more than 99% of the cells were positively stained. Data are representative of three independent experiments. (c) Detection of CFSE-labeled HUCPVCs in the lungs by flow cytometry. CFSE-labeled HUCPVCs (1 × 10^6^ cells) were administered via the pulmonary artery as described in Materials and Methods (Section 2.2). At the end of the experiment, samples from the upper, middle, and lower sections of the left lung were obtained and analyzed by flow cytometry. Data are representative of three independent experiments. (d) Identification of HUCPVCs in the lungs by immunostaining against human vimentin. Representative images of lung tissue sections obtained at the end of the perfusion from the lungs receiving infusion of vehicle (A) or HUCPVCs (B) at ×400 original magnification (inset: ×1000 original magnification). Black arrows indicate vimentin-positive cells. Please note that vimentin-positive cells show a diffuse cytoplasmic staining.

First, we analyzed the immunophenotypic characteristics of the HUCPVCs used in this study to determine whether they conformed to the minimal criteria defining MSCs [25]. HUCPVCs highly expressed (>98%) the stromal determinants CD44, CD90, and CD105 and were negative (<1%) for monocyte/macrophage (CD11b), endothelial (CD34), and hematopoietic (CD45) markers (Figure 1). To test the beneficial effects of MSC therapy during donor lung preservation, we infused the HUCPVCs via the pulmonary artery during the procurement of the lungs from DCD (after 1 h of cardiac arrest). The period of warm ischemia was extended to 2 h before cold preservation with a low potassium dextran solution (Perfadex). Finally, lungs were perfused with Steen solution alone for 1 h using the EVLP technique in order to acquire functional data (Figure 2). To assess the retention of HUCPVCs in the lung parenchyma, a series of experiments (*n* = 3) were carried out using CFSE-labeled cells (Figure 3). More than 99% of the HUCPVCs were effectively stained with CFSE as it can be seen by fluorescence microscopy and flow cytometry analysis (Figures 3(a) and 3(b)). The administration of 1 × 10^6^ CFSE-labeled HUCPVCs was associated with the detection of CFSE-positive cells in the lungs at the end of the experiments (Figure 3(c)). The majority of the HUCPVCs were located in the upper and middle sections of the lungs, possibly due to the route of administration (via the pulmonary artery) and the rapid retention of the human cells in the rat microvasculature because of their size (Figure 3(c)). In fact, the presence of large cells in the microvasculature was observed only in hematoxylin-eosin-stained lung sections from the cell therapy group (Figure S2). To further identify HUCPVCs in the lungs, we performed an immunohistochemical analysis using anti-human vimentin to specifically stain human donor cells. We found that HUCPVCs, evidenced as large cells with a strong-diffuse cytoplasmic staining, were mainly retained in the lung microvasculature (Figure 3(d)). Vimentin-positive cells were not detected in lungs receiving vehicle.

### 3.2. Effect of HUCPVCs on Donor Lung Inflammation and Oxidative Stress Parameters during Organ Preservation

**Figure 4:**
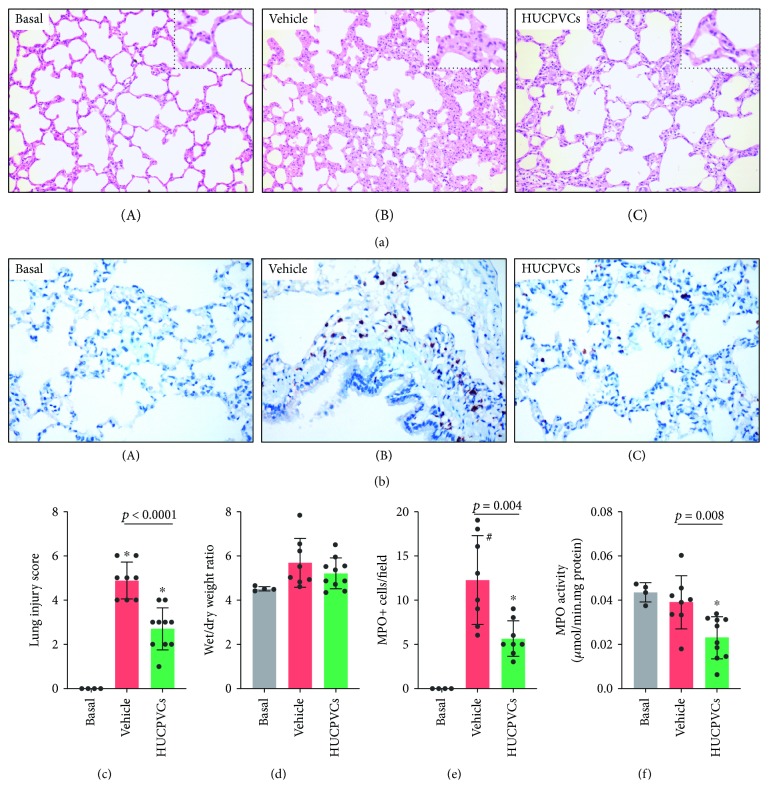
Lung injury and inflammation during organ preservation. (a) Hematoxylin and eosin stained sections obtained at the end of the perfusion from the lungs receiving infusion of vehicle (B) or HUCPVCs (C). Lung sections from untouched rats (basal, (A)) were used as controls. Alveolar septal thickening and interstitial cellular infiltration were more evident in the lungs from the vehicle group. Results show representative images from each group at ×200 original magnification (inset: ×400 original magnification). (b) Representative images of the lung tissue sections with immunostaining against myeloperoxidase (MPO) at ×400 original magnification. MPO-positive cells appear brown. (c) Histological injury scores of the lungs in different groups were quantified as described in Materials and Methods. Data are expressed as mean ± S.D. ^∗^ *p* < 0.0001 against the basal group derived from one-way ANOVA after multiple comparisons Tukey post hoc test. (d) Lung wet/dry weight ratios at the end of the perfusion. Data are expressed as mean ± S.D. (e) Quantitation of MPO-positive cells (from (b)). MPO-positive cells were counted in 10 visual fields/section at ×200 magnification, and the average number for each sample was calculated. Data are expressed as mean ± S.D. ^∗^ *p* < 0.05 and ^#^ *p* < 0.0001 against the basal group derived from one-way ANOVA after multiple comparisons Tukey post hoc test. (f) Lung myeloperoxidase (MPO) activity. Data are expressed as mean ± S.D. ^∗^ *p* < 0.01 against the basal group derived from one-way ANOVA after multiple comparisons Tukey post hoc test.

Each phase of lung procurement generates different degrees of organ injury that accumulate throughout the entire procedure. The total amount of organ damage directly correlates with the subsequent incidence of PGD. In our experimental setting, most of the organ damage occurred during the period of warm ischemia which was extended for up to 2 h. For convenience, we decided to infuse the cell therapy after 1 h of warm ischemia. Thus, donor lungs accumulated a certain degree of injury that allowed us to determine whether HUCPVCs can prevent further damage. Microscopic assessment of histopathologic lung injury at the end of the experiments showed that lungs receiving vehicle exhibited characteristic signs of inflammatory damage including widespread alveolar wall thickening, mild interstitial edema, and the presence of cellular infiltrates in both the interstitium and the alveoli (Figure 4(a)). By comparison, lungs receiving HUCPVCs also showed alveolar thickening and mild edema, but signs of inflammation were less evident as the tissue histology was more similar to untouched rat lungs (Figure 4(a)). In addition, immunohistochemical analysis for the detection of myeloperoxidase (MPO), an enzyme contained in primary granules of cells of the myeloid lineage (neutrophils in particular), demonstrated the presence of MPO-positive cells in the alveolar wall (Figure 4(b)). As quantified in Figure 4(c), the lung tissue in the vehicle group exhibited a significant increase in the histological injury score compared with untouched rat lungs (basal), whereas the administration of HUCPVCs significantly improved the lung histological injury score compared with the vehicle group (2.7 ± 0.9 vs. 4.9 ± 0.8, respectively; *p* < 0.0001). The formation of pulmonary edema was assessed by lung wet/dry weight ratios. No significant differences were observed in lung wet/dry weight ratio between HUCPVC- and vehicle-treated groups (5.2 ± 0.7 vs. 5.7 ± 1.1, respectively; *p* = n.s.), and values for wet/dry weight ratios in both groups were slightly higher than those found for untouched rat lungs (Figure 4(d)). The absence of edema may be related to the use of Steen solution for the perfusion of the lungs, which contains human albumin to maintain an optimal oncotic pressure that reduces the formation of pulmonary edema during perfusion [31]. The number of MPO-positive cells retained in the interstitium and in the alveolar wall was significantly lower in the HUCPVC group when compared with the vehicle group (6 ± 2 vs. 12 ± 5 MPO+ cells/field, respectively; *p* = 0.004) (Figure 4(e)). To confirm the histological observations, we quantified neutrophil retention in the lung tissue by measuring total MPO activity. As shown in Figure 4(f), the infusion of HUCPVCs significantly decreased MPO activity by 41% compared with the vehicle group (0.023 ± 0.009 vs. 0.039 ± 0.011*μ*mol/min·mg protein, respectively; *p* = 0.008). Most of the neutrophils were retained in lungs receiving vehicle as MPO values were similar to those found in basal rat lungs without perfusion (Figure 4(f)). These data imply that HUCPVC therapy was effective in reducing the inflammatory response triggered by ischemia, during the preservation of donor lungs.

**Figure 5:**
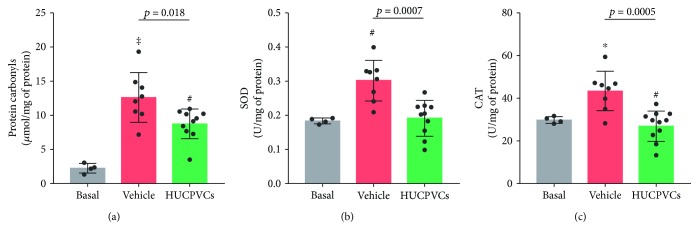
Effect of HUCPVCs on oxidative stress parameters during the preservation of donor lungs. Lung biopsies were obtained at the end of the perfusion from the lungs receiving infusion of vehicle or HUCPVCs. Lung samples from untouched rats (basal) were used as controls. Protein lysates were prepared and assayed as described in Materials and Methods. (a) Protein carbonyl content. (b) SOD activity. One unit of SOD is defined as the amount of enzyme that inhibits the reduction of NBT by 50% under assay conditions. (c) CAT activity. One unit of CAT is defined as the amount of enzyme which breaks down 1 nmol of H_2_O_2_ per min under assay conditions. Data are expressed as mean ± S.D. ^∗^ *p* < 0.05, ^#^ *p* < 0.01, and ^‡^ *p* < 0.001 against the basal group derived from one-way ANOVA after multiple comparisons Tukey post hoc test.

ROS overproduction can damage all types of biological molecules, and carbonyl groups are the major products of ROS-mediated oxidation reactions. Consequently, protein carbonyl groups have been widely used as biomarkers of oxidative stress because of their relative early formation and stability [32]. As expected, we found that protein carbonyl content significantly increased by 5.6-fold in lungs from the vehicle group when compared with untouched rat lungs (Figure 5(a)). Moreover, the activities of the antioxidant enzymes SOD and CAT increased by 64% and 46% in the vehicle group with respect to untouched rat lungs (basal), respectively (Figures 5(b) and 5(c)). The upregulation of SOD and CAT indicates that the cellular antioxidant enzyme system responded to the oxidative damage in order to restore ROS homeostasis. These data demonstrate that oxidative stress occurred during the preservation of donor lungs in our experimental setting. Interestingly, cell therapy with HUCPVCs significantly decreased the content of carbonylated proteins in the lungs when compared with the vehicle group (Figure 5(a)). However, the level of carbonylated proteins in lungs from the HUCPVC group was significantly higher than in untouched rat lungs (basal). Most of these protein carbonyl groups may be generated during the first hour of the warm ischemia period, before the infusion of the cells. In agreement with this notion, the administration of HUCPVCs was able to prevent the increase in the activities of SOD and CAT implying that the overproduction of ROS was controlled by the therapy (Figures 5(b) and 5(c)).

### 3.3. Effect of HUCPVCs on Donor Lung Function

**Figure 6:**
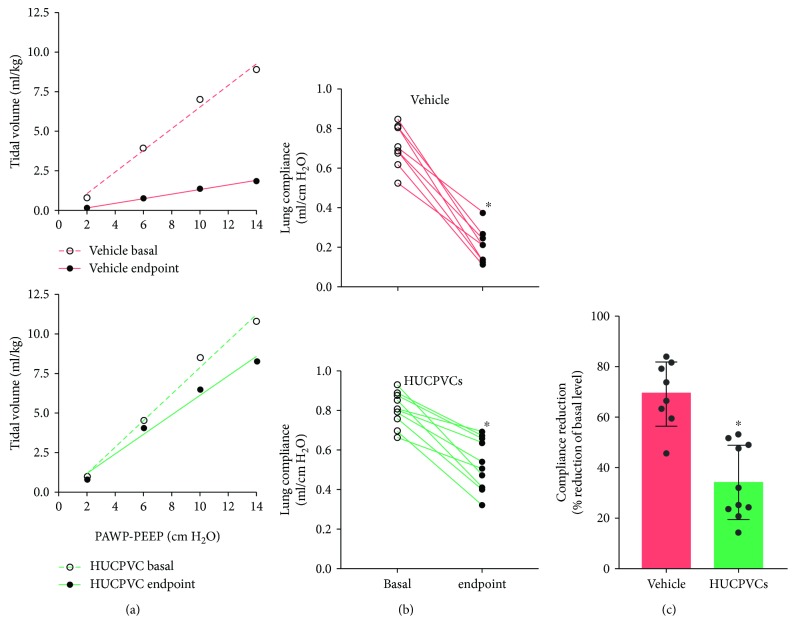
Lung functional data. (a) Pressure-volume curves in mechanically ventilated lungs. Curves were generated by using sequential pressure steps in which the tidal volume reached at each step was measured by the ventilator. Open symbols represent basal measurements, and closed symbols represent measurements obtained at the end of the perfusion. Graphs show a baseline vs. endpoint pressure-volume curve for a representative lung from vehicle and HUCPVC groups. The slope of the pressure-volume curve represents the lung compliance. (b) Individual changes in lung compliance for vehicle and HUCPVC groups. Lung compliance was determined at baseline (open symbols) and at the end of the perfusion (closed symbols) from pressure-volume curves data. ^∗^ *p* < 0.0001 vs. basal derived from paired *t* test. (c) Lung compliance reduction expressed as percentage with respect to baseline. ^∗^ *p* < 0.0001 derived from unpaired *t* test.

We next evaluated the effect of the HUCPVC therapy in donor lung function. It has been recently reported that, in the clinical setting, lung compliance and ventilatory pressures are important parameters to evaluate graft quality after EVLP in both controlled and uncontrolled DCD [33, 34]. Lung compliance refers to the magnitude of change in lung volume as a result of the change in pulmonary pressure. Here, we determined the pressure-volume curve to assess the mechanical properties of donor lungs (Figure 6(a)). Lung compliance was obtained from the slope of this pressure-volume curve. To avoid biological differences between animals, we compared the compliance of each individual lung between baseline and the end of the perfusion period (Figure 6(b)). We found that lung compliance was significantly reduced by 69% and 34% in the vehicle group and HUCPVC group, respectively (Figure 6(c)). Of note, HUCPVC therapy significantly reduced by 50% the decrease in lung compliance related to the procedure. These data indicate that the antioxidant and anti-inflammatory effects of MSCs led to a better preservation of the donor lung function throughout the entire procedure.

## 4. Discussion

Currently, the main clinical practice for donor lung preservation is static cold storage. This procedure is mainly based on reducing cell metabolism by storing the lungs at 4°C for an acceptable ischemic time of less than 6 h [12]. Sterile inflammatory processes occur during this period of anoxic ischemia and also at the time of organ reperfusion (i.e., IRI). Increased formation of ROS is involved in the development of lung injury through the activation of nuclear factor-kappa B (NF-*κ*B) which precedes the release of proinflammatory cytokines [35]. Accordingly, lung preservation solutions that reduce ROS production (i.e., low-potassium dextran) are of choice because they have shown to decrease the incidence of PGD [36, 37]. In this study, we demonstrated that the administration of HUCPVCs during the warm ischemia period prevents the development of oxidative stress and, hence, better preserves donor lung function. Our results suggest that HUCPVC therapy at the moment of organ ablation may represent a novel strategy to improve donor lung preservation.

One of the major attributes of MSCs is their anti-inflammatory potential, which is mainly exerted through a paracrine mechanism. A previous report found that i.v.-infused MSCs are activated in the lungs to secrete anti-inflammatory molecules which reduce infarct size in a mouse model of acute myocardial infarction [38]. More recently, we demonstrated that both BM-MSCs and HUCPVCs can mediate the switch from proinflammatory to anti-inflammatory macrophages at the infarct site [39]. Moreover, anti-inflammatory exosomes derived from MSCs prevent LPS activation of RAW 264.7 macrophages and also suppress LPS-induced inflammation in mice [40]. In this context, cell therapy using MSCs has demonstrated therapeutic potential to prevent pulmonary IRI and to recover damaged lungs during EVLP [15, 16, 19]. However, the potential benefit of MSC therapy on donor lung preservation has not been previously studied. For this reason, in the present study, and with the aim of maximizing donor lung availability, we infused HUCPVCs during donor lung ablation to better preserve lung function. An important evidence of pulmonary physiological dysfunction in the clinical setting is decreased lung compliance [41], which depends on the elastin and collagen fibers present in the lung parenchyma and the alveolar surface tension. Low lung compliance is a characteristic of patients with acute respiratory distress syndrome or pulmonary fibrosis. Typically, lung compliance decreases during harvesting, preservation, and transplantation of the donor organ [41]. Noteworthy, HUCPVC administration was able to significantly reduce the loss of lung compliance during organ preservation. This result has an important implication in the clinical setting, as it may increase the number of suitable lungs available for transplantation.

There are multiple steps in the transplantation procedure that may injure the donor lung. Organ damage can occur before or as a consequence of donor death, during the ischemic period or during reimplantation and reperfusion in the recipient. In an IRI rat model, it has been demonstrated that hypoxic MSCs can attenuate inflammation by infusing them a few minutes before the ischemic insult [19]. Moreover, Stone et al. [16] described that MSCs were more effective than their extracellular vesicles for restringing lung inflammation when administered via PA before ischemia and also facilitated damaged organ repairing when used during EVLP. In line with this, Mordant et al. [15] demonstrated that MSC therapy was very promising for reconditioning donor lungs by enhancing the repairing potential of EVLP. Of note, the main difference among these studies and ours is that they were designed with the aim of repairing unacceptable lungs while, in contrast, we aimed to preserve donor lung functionality by limiting the ischemic injury.

It is well known that ROS are key players and initiators of lung IRI as the administration of antioxidant compounds or antioxidant enzymes, such as SOD and CAT, can prevent tissue damage during reperfusion [35, 42]. In addition, ROS function as intracellular signaling molecules; thus, regulation of ROS homeostasis by antioxidant enzymes is important to trigger redox-specific responses [43]. In this regard, it has been demonstrated that MSCs are resistant to oxidative stress because they constitutively express high levels of antioxidant enzymes and can effectively scavenge ROS [44]. It was also reported that the anti-inflammatory effect of MSCs in a model of renal IRI is mediated by suppression of oxidative stress [45]. Hence, the therapeutic effect of MSCs may be related, at least in part, to their potential to control oxidative insults preventing tissue damage. In this way, HUCPVC administration during donor lung ablation may inhibit the development of inflammation by blocking ROS-activated signaling pathways. In fact, here, we found that HUCPVCs significantly reduced the recruitment of marginated neutrophils into the interstitium and the alveolar wall, as demonstrated by a lower number of MPO-positive cells and a lower MPO activity in the lungs. It is well known that the prolonged neutrophil transit time through the lung vessels contributes to the formation of marginated neutrophil pools [46]. During ischemia, neutrophils are recruited from circulation or marginated pools to the injured lung. In our experimental setting, the oxidative stress triggered during the warm ischemia period may drive the retention of marginated neutrophils in the lung tissue. Contrarily, in HUCPVC-treated lungs, which were protected against oxidative stress, the majority of donor neutrophils were not retained and were washed from the lungs during the perfusion.

In conclusion, developing new strategies to improve donor lung preservation may solve the current organ shortage by increasing the number of lungs considered acceptable for transplantation. Anti-inflammatory gene or stem cell therapies demonstrated great efficacy to reduce IRI and to recover injured lungs by EVLP. Unlike previous studies, here, we enhanced the preservation of lungs obtained from DCD by injecting MSCs during warm ischemia. We have established that MSCs protect against oxidative stress and also prevent alveolar wall thickening and neutrophil recruitment. These data infer that MSCs were able to avoid lung damage by inhibiting ROS-mediated inflammatory responses. Most importantly, donor lung function was significantly conserved in the lungs receiving MSC therapy. Accordingly, the implementation of MSC therapy during lung ablation may have a great clinical impact, as it not only could be accomplished in small centers, which are unlikely to establish their own clinical EVLP program, but also it would improve organ preservation during the whole procurement procedure.

## Data Availability

The data used to support the findings of this study are available from the corresponding author upon request.

## Supplementary Materials

Supplementary Materials Figure S1. Mechanical ventilation. (a) Real-time monitoring of the ventilatory parameters and pressure curves on the digital display of the ventilator (SomnoSuite™ Small Animal Anesthesia System, Kent Scientific). The upper half of the screen displays the value of the parameters which are being measured. The lower half of the screen displays a graph of the data. (b) Graphical representation of pressure vs. time curves in different respiratory cycles. Real-time data was collected in a computer through the USB port for further analysis. Abbreviations: Vt: tidal volume; Ppeak: peak pressure; PEEP: positive end-expiratory pressure. Figure S2. Hematoxylin and eosin stained sections obtained at the end of the perfusion from the lungs receiving infusion of vehicle (left panels) or HUCPVCs (right panels). Representative images from each group at ×400 original magnification. Black arrows indicate the presence of large cells in the lung microvasculature. Supplementary methods: the surgical procedure. Supplementary Video 1. Showing the lung during ventilation and perfusion with Steen solution.
