# Supplementary material for: Mesenchymal Stem Cell Therapy Facilitates Donor Lung Preservation by Reducing Oxidative Damage during Ischemia
Source: Stem Cells Int. 2019 Aug 5;2019:8089215. doi: 10.1155/2019/8089215 (PMC6701419; doi:10.1155/2019/8089215)

Mesenchymal stem cells therapy facilitates donor lung preservation by reducing oxidative-damage during ischemia.

Supplementary Methods: surgical procedure.

1. Intrahepatic administration of Heparin (1000 U/kg). After 5 min, euthanasia was carried out using sodium thiopental (250 mg/kg) administered by the same route.

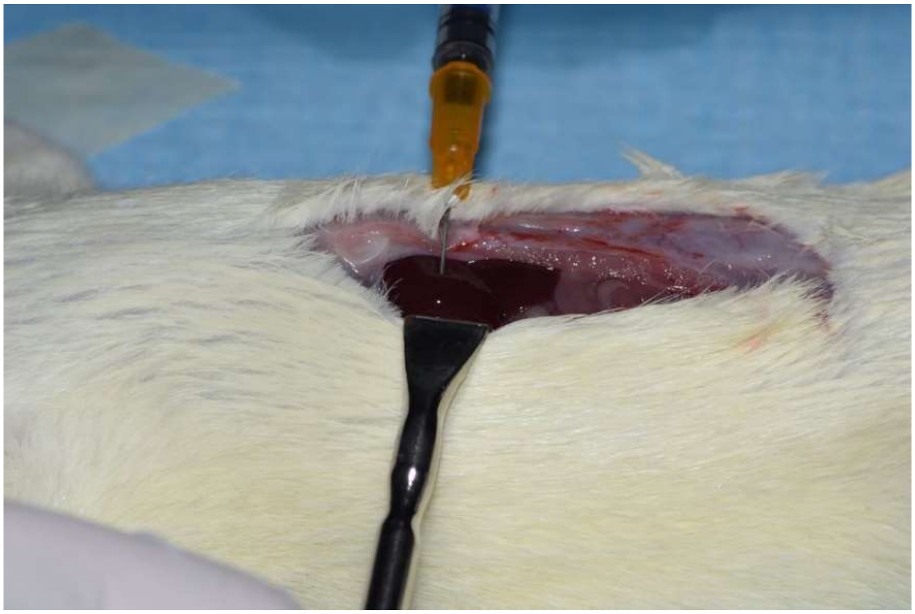

2. Intubation by tracheotomy.

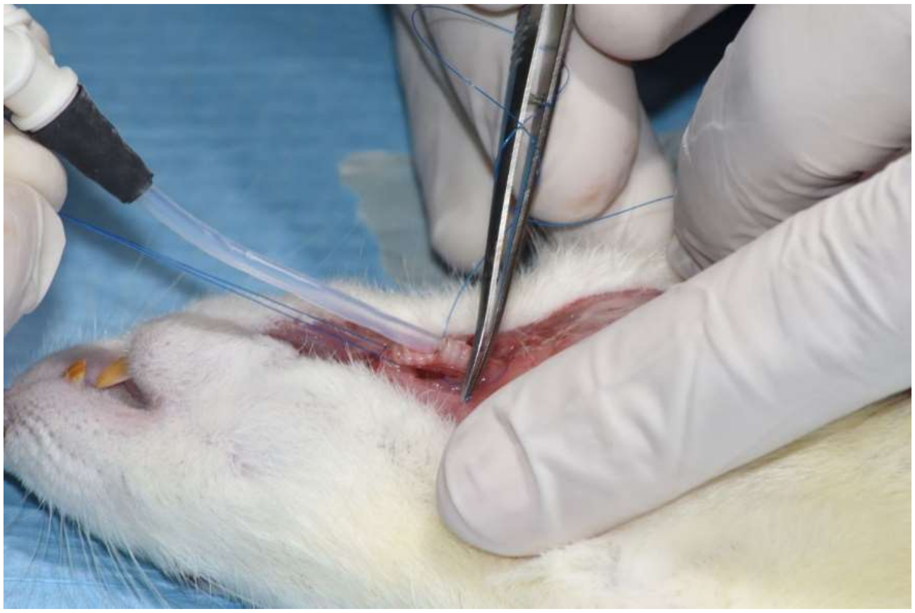

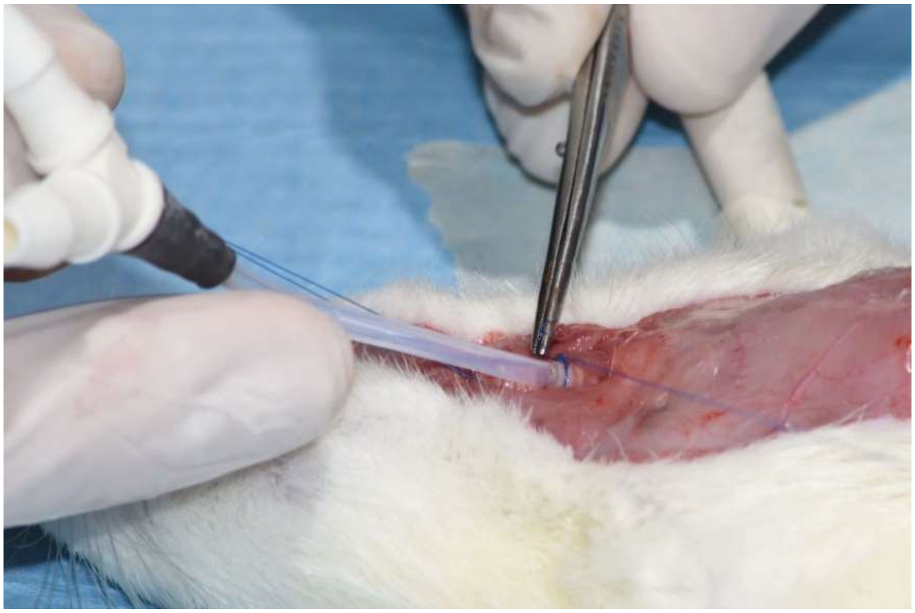

3. Mechanical ventilation and median sternotomy.

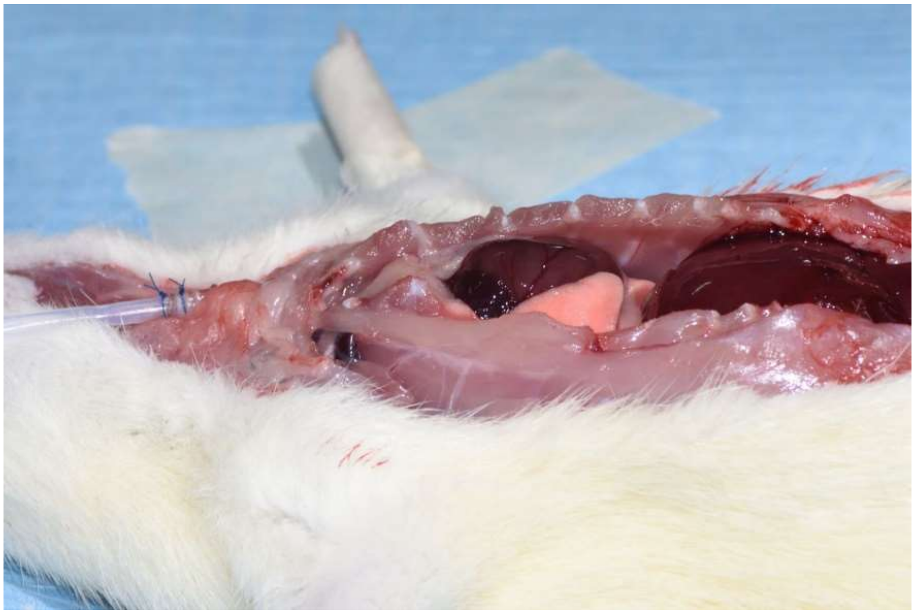

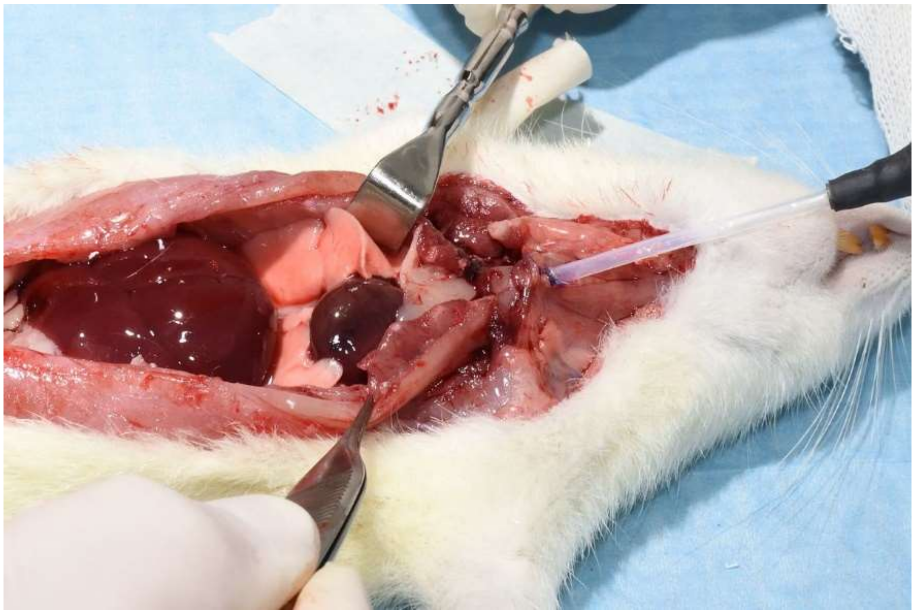

4. Placement of the cannula in the pulmonary artery through the right ventricle.

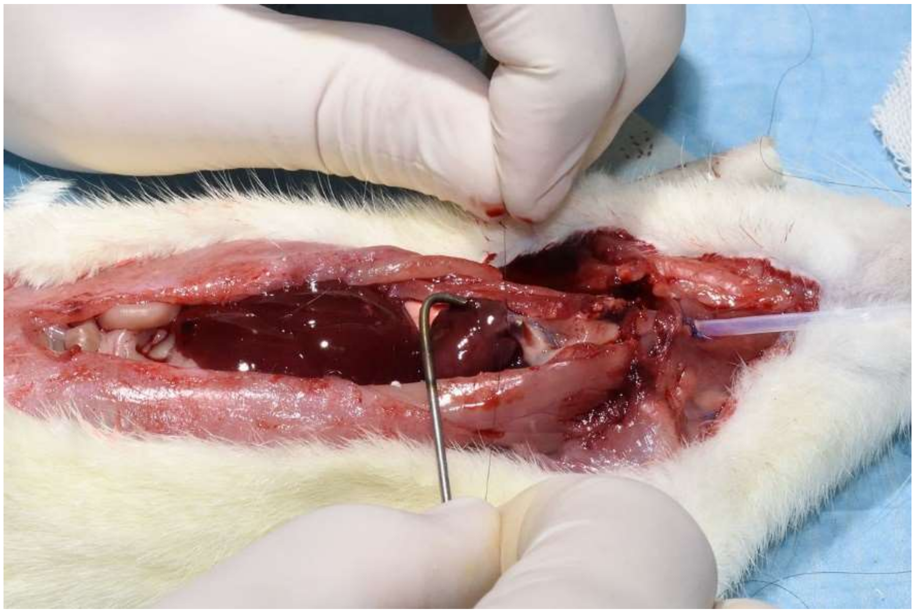

5. Ablation of the lungs.

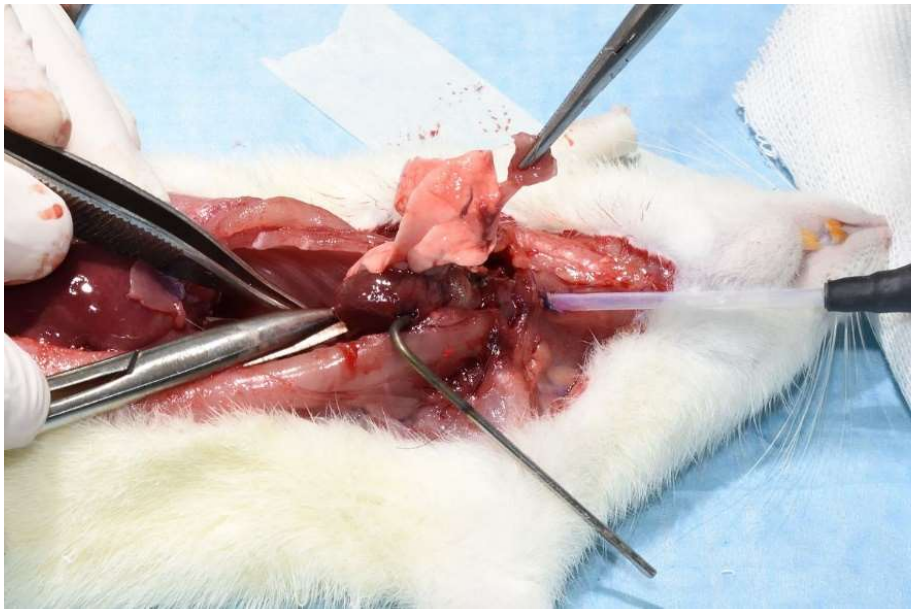

6. Washing with physiological solution. Note the placement of the cannula in the pulmonary artery.

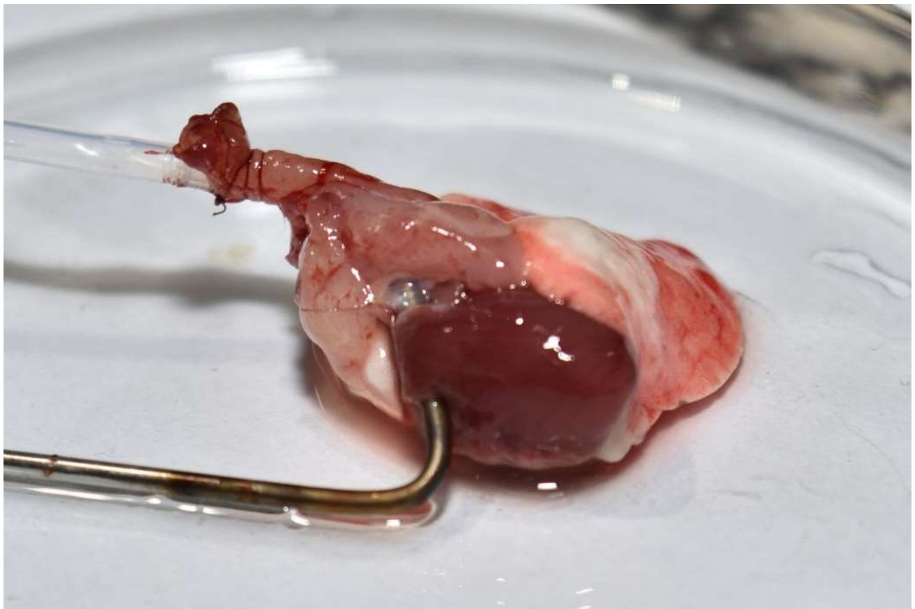

7. Positioning of the lungs for the administration of cells and reperfusion.

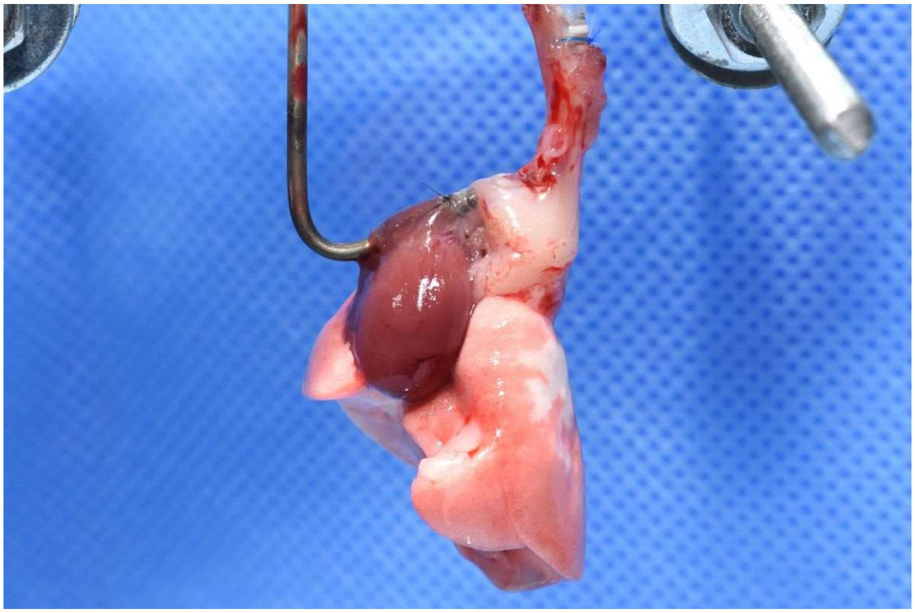

Supplement: Supplementary Materials — Figure S1. Mechanical ventilation. (a) Real-time monitoring of the ventilatory parameters and pressure curves on the digital display of the ventilator (SomnoSuite™ Small Animal Anesthesia System, Kent Scientific). The upper half of the screen displays the value of the parameters which are being measured. The lower half of the screen displays a graph of the data. (b) Graphical representation of pressure vs. time curves in different respiratory cycles. Real-time data was collected in a computer through the USB port for further analysis. Abbreviations: Vt: tidal volume; Ppeak: peak pressure; PEEP: positive end-expiratory pressure. Figure S2. Hematoxylin and eosin stained sections obtained at the end of the perfusion from the lungs receiving infusion of vehicle (left panels) or HUCPVCs (right panels). Representative images from each group at ×400 original magnification. Black arrows indicate the presence of large cells in the lung microvasculature. Supplementary methods: the surgical procedure. Supplementary Video 1. Showing the lung during ventilation and perfusion with Steen solution. [file 8089215.f1.zip › Supplementary Methods. Surgical Procedure.pdf]
